# Targeting of the intracellular redox balance by metal complexes towards anticancer therapy

**DOI:** 10.3389/fchem.2022.967337

**Published:** 2022-08-11

**Authors:** María Isabel Murillo, Christian Gaiddon, Ronan Le Lagadec

**Affiliations:** ^1^ Instituto de Química, Universidad Nacional Autónoma de México, Ciudad Universitaria, Ciudad de México, Mexico; ^2^ Strasbourg Université, Inserm UMR_S U1113, IRFAC, Strasbourg, France

**Keywords:** anticancer therapy, oxidoreductases, redox balance, transition metals, tumor metabolism

## Abstract

The development of cancers is often linked to the alteration of essential redox processes, and therefore, oxidoreductases involved in such mechanisms can be considered as attractive molecular targets for the development of new therapeutic strategies. On the other hand, for more than two decades, transition metals derivatives have been leading the research on drugs as alternatives to platinum-based treatments. The success of such compounds is particularly due to their attractive redox kinetics properties, favorable oxidation states, as well as routes of action different to interactions with DNA, in which redox interactions are crucial. For instance, the activity of oxidoreductases such as PHD2 (prolyl hydroxylase domain-containing protein) which can regulate angiogenesis in tumors, LDH (lactate dehydrogenase) related to glycolysis, and enzymes, such as catalases, SOD (superoxide dismutase), TRX (thioredoxin) or GSH (glutathione) involved in controlling oxidative stress, can be altered by metal effectors. In this review, we wish to discuss recent results on how transition metal complexes have been rationally designed to impact on redox processes, in search for effective and more specific cancer treatments.

## Introduction

Reduction-oxidation (redox) processes are at the center of many functions in chemistry and biology and have become one of the leading research topics in biochemistry and biophysics. (Monteiro and Stern, 1996; Nakamura et al., 1997; Berglund et al., 2002; Guiseppi-Elie et al., 2002; Finkel, 2003; Della et al., 2011). Redox proteins and enzymes can also conduct reactions of industrial and pharmaceutical importance. (Truppo, 2017; Prier and Kosjek, 2019). The fundamental structure of such proteins consists of catalytic sites connected by redox chains, which can be described as multielectron redox centers or clusters of single electron redox centers that interact with substrates and function as sources or sinks of electrons. Most transition metals can display multiple oxidation states and can be found as active sites of many proteins and, as such, playing essential roles in oxidoreduction functions (Sheldon and Woodley, 2018; Turner and Kumar, 2018). Oxidoreductases are considered catalysts for important biological processes that require electron transfers, including photosynthesis, respiration, metabolism, and signaling processes governing gene regulation and expression (Allen et al., 1995). Between 30 and 50 percent of all proteins are considered oxidation/reduction enzymes or metalloproteins. As such, studies aimed at elucidating the molecular and electrochemical properties linked with the chemical and biological electron transport systems displayed by redox proteins have been extensively developed (Prabhulkar et al., 2012).

Redox homeostasis is maintained by the net physiologic balance between reducing and oxidizing equivalents within subcellular compartments, in particular through components like reactive oxygen species (ROS) and antioxidant enzymes (Berglund et al., 2002). Studying and understanding such processes is fundamental for cancer treatment (Narayanan et al., 2020). Traditionally, the free-radical theory of cancer considered that oxidative stress due to reactive oxygen/nitrogen species (ROS/RNS) could generate DNA damage and promote genetic instability (Hussain et al., 2003). However, ROS/RNS are now thought to be involved in not only in direct DNA damage but also in modulations of redox-regulated signaling pathways, which may be both beneficial or detrimental in cancers.

**FIGURE 1:**
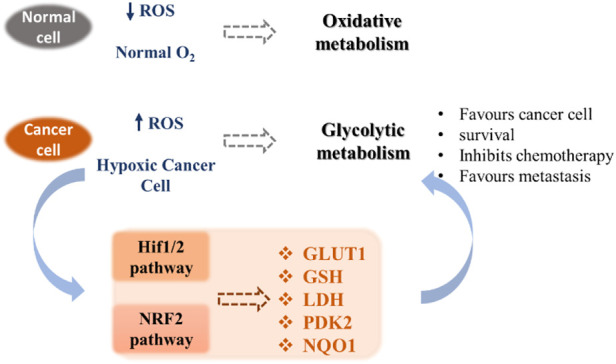
Metabolic pathways involved in tumor adaptation to its stressful environment (Gaiddon et al., 2021).

Unlike normal differentiated cells, which rely primarily on mitochondrial oxidative phosphorylation to generate the necessary energy in the form of ATP for cellular processes, most cancer cells rely on aerobic glycolysis. After tumor growth, there are fewer blood vessels, which leads to less oxygen (hypoxia), and cancer cells develop a hypoxic response through the hypoxia-responsive transcription factor HIF1A. This transcription factor plays a key role by inducing the expression of VEGF, a growth factor that stimulates vascularization and the expression of glucose transporters such as GLUT1 and redox enzymes (for instance LDH). This allows the reprogramming of metabolism towards glycolytic metabolism, which does not require as much oxygen. This process is called the Warburg effect (Vaupel and Multhoff, 2021). Cancer cells also exhibit increased ATP production and important levels of ROS, which permits to maintain high cell proliferation through the metabolic resetting. Antioxidant therapy can protect normal cells by activating cell survival signaling cascades, such as the nuclear factor erythroid 2-related factor Nrf2 pathway (Irwin et al., 2013). Nrf2 is a crucial regulator that protects cells from oxidative stress. Adaptations resulting from Nrf2 activation may have beneficial effects under stress conditions through modulation of antioxidant pathways but may also participate in the development of resistance to cancer therapy (Zhang, 2010). Due to their implications in cancer pathogenesis, redox homeostasis and the metabolic switch from glycolysis to oxidative phosphorylation appear as promising targets for cancer therapy (Gaiddon et al., 2021). These pathways include HIF1/2 and NRF2 mechanisms that contribute to the modification of the expression of transporters (*e.g*. glucose transporters), redox enzymes (*e.g*. LDH, PDK2), chaperone proteins and antioxidant enzymes (*e.g*. GSH) (Figure 1).

**FIGURE 2:**
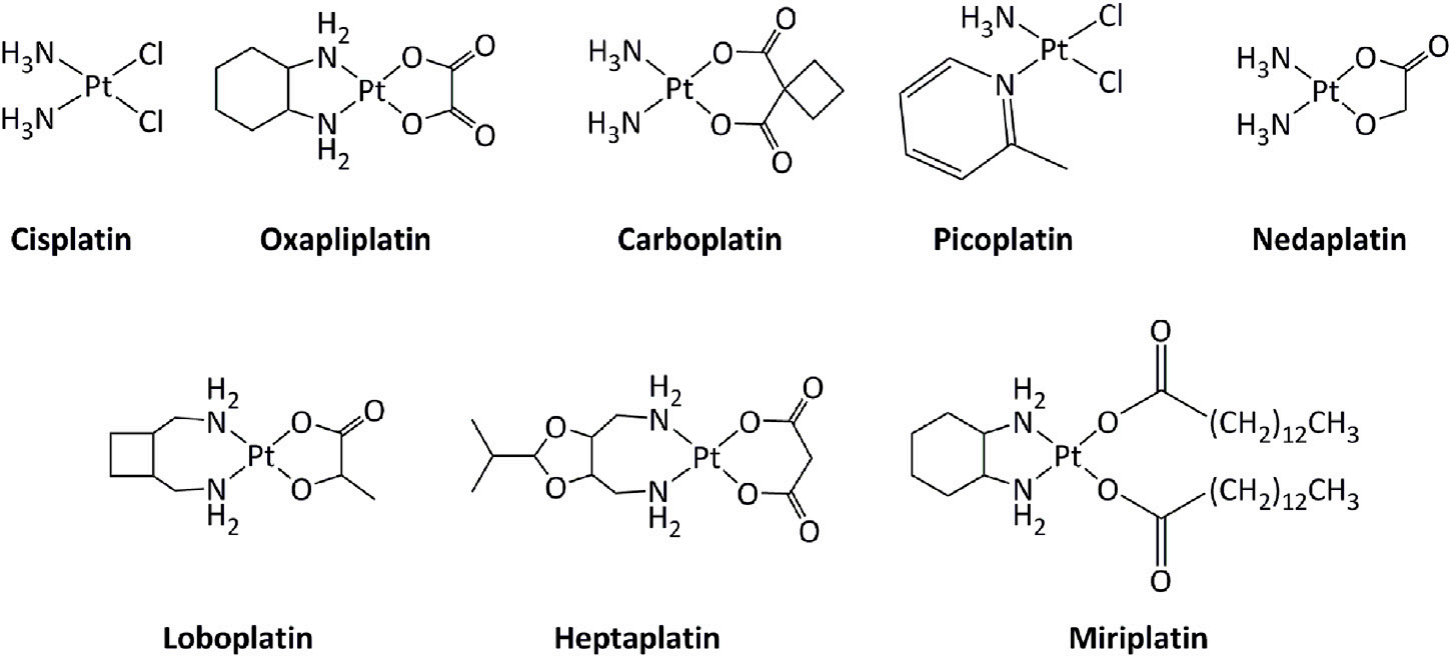
Platinum complexes used as anticancer drugs.

Transition metal-based derivatives have been intensively studied for their attractive anticancer properties (Raymond et al., 1998; Garbutcheon et al., 2011; Ndagi et al., 2017; Parveen, 2020). Platinum-based drugs, mainly cisplatin and its analogs carboplatin and oxaliplatin (Figure 2), have been used worldwide in cancer treatment (Dilruba and Kalayda, 2016). Other platinum-based molecules, such as miriplatin, nedaplatin, lobaplatin, and heptaplatin have also been approved regionally. The mode of action of these compounds is mostly through direct interactions with DNA, inducing DNA damage, which activates series of molecular mechanisms, including induction of the p53 tumor suppressor gene. Consequently, alterations in the p53 pathway, such as mutations in p53, lower the response toward platinum-based drugs (Blanchet et al., 2021). Additionally, the low selectivity of platinum drugs for cancer cells generates serious side effects on various tissues, including the nervous system and the muscles (Benosman et al., 2007; Benosman et al., 2011; Oun et al., 2018; Voisinet et al., 2021).

**FIGURE 3:**
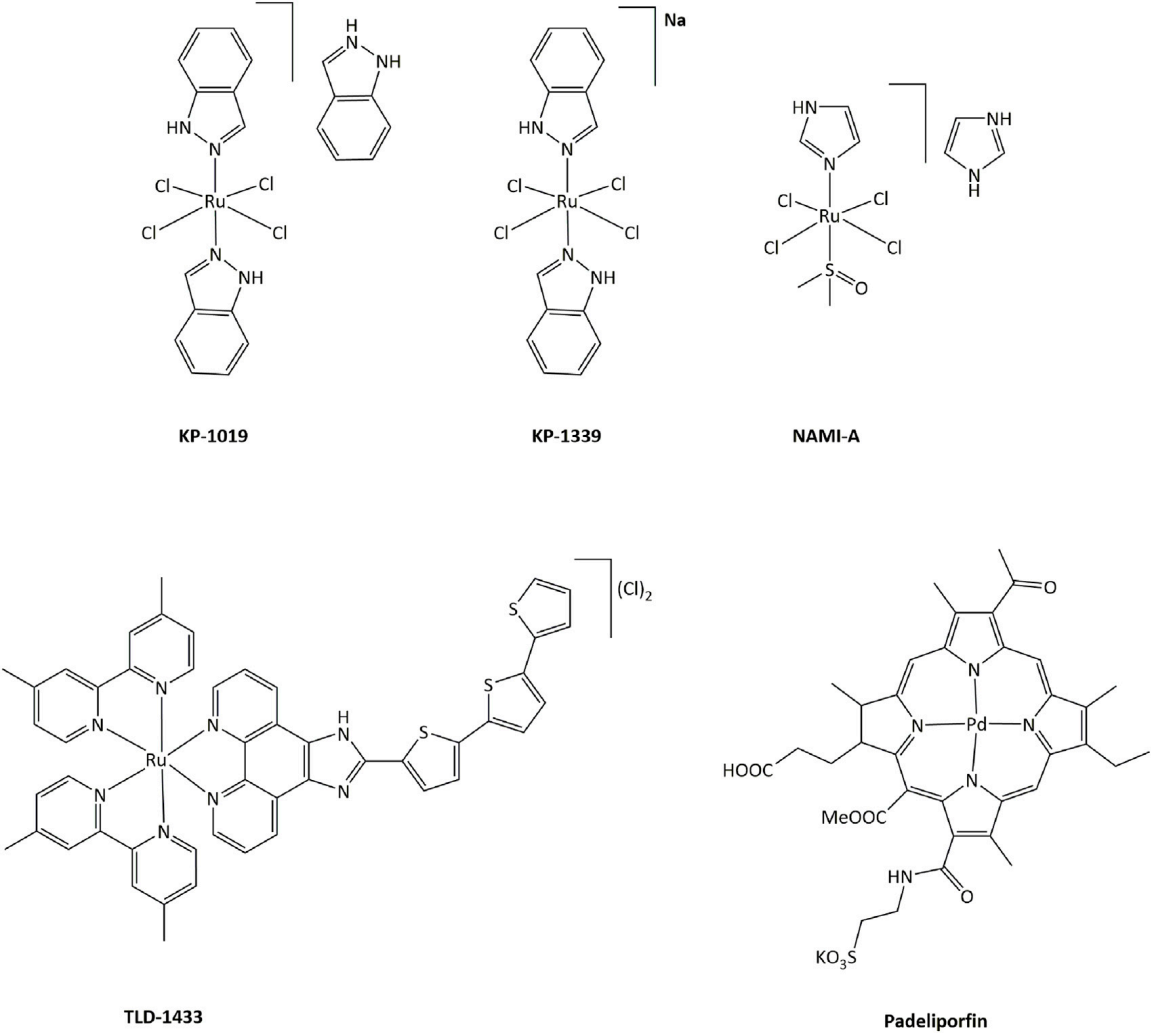
Ruthenium complexes studied in clinical trials as candidates for anticancer treatments and palladium compound approved for clinical use.

In order to limit such severe side-effects caused by platinum compounds, the use of other metals have been extensively explored. Both the redox properties of the metal and of the ligands in transition metal complexes can generate new routes of action that can bypass resistance mechanisms toward platinum or other DNA damaging drugs. Ruthenium derivatives have often been shown to exhibit a lower toxicity, linked with a higher selectivity towards cancer cells, than platinum-based drugs (Oun et al., 2018). In particular, four ruthenium derivatives have been evaluated clinical trials. **NAMI-A** was successfully studied in phase I, but poor efficacy was obtained in phase II, while the low solubility of related compound **KP1019** limited further development (Figure 3). (Alessio and Messori, 2019; Bergamo and Sava, 20072007; Baier et al., 2022; a; Monro et al., 2019)

Ruthenium(III) **KP-1339** is currently undergoing clinical trials, delivering promising Ib phase data for anticancer activity. Ruthenium(II) complex **TLD-1433** acts as a photosensitizer, and is currently being evaluated in phase II for photodynamic therapy (PDT) against human non-muscle invasive bladder cancer. 1) Furthermore, many other transition metal complexes have been studied for their cytotoxic properties and potential use as anticancer drugs (Meier-Menches et al., 2018; Zamora et al., 2018; McFarland et al., 2019; Anthony et al., 2020). Notably, palladium-based compound **Padeliporfin** (commercially known as **Tookad**, Figure 3) has recently been approved by the European Medicines Agency in the European Union for PDT in patients with low-risk prostate cancer (Coleman et al., 2021).

The mechanisms of action for metal complexes can be varied, and in particular can be driven by redox reactions. In 2011, Heffeter *et al.* reviewed how metal complexes could carry out their cytotoxic activity in cancer cells through interactions with the cellular redox homeostasis (Jungwirth et al., 2011). A review by Sadler *et al.* in 2013 discusses multiple targets of metal complexes that are able to interfere with the cellular redox state (Romero-Canelón and Sadler, 2013). Redox-based mechanisms have also been successfully exploited, particularly with Ru(III) and Pt(IV) derivatives, in processes where the complexes act as prodrugs that are activated by the reducing environment of cancer cells, as highlighted in a 2012 review by Lippard *et al.* and in another review by Sadler *et al.* in 2017 (Graf and Lippard, 2012; Zhang and Sadler, 2017). Catalytic action has also been discussed for ruthenium and iridium compounds (Dougan et al., 2008; Liu and Sadler, 2014). More recently, one review focused on how metal-based drugs could induce anticancer immune responses, and another on transition metal complexes for photodynamic therapy (PDT) and photoactivated chemotherapy (PACT). (Englinger et al., 2019; Imberti et al., 2020).

Within this context and because of the high number of studies published every year on the anticancer activity of transition metal complexes, in this review, we wish to present updated information that highlight the importance of redox processes in cancer metabolic pathways, and how tumor development may be hindered by redox interactions with metal complexes. In addition to platinum and ruthenium compounds, we will discuss representative and recent examples of iron, osmium, iridium, rhodium, copper, silver and gold complexes that show redox-mediated anticancer activity.

## The redox landscape in cancer

The redox balance is efficiently regulated in living organisms. For instance, ROS and RNS are generated during normal physiological metabolism and in response to stress, including exposure to xenobiotics, cytokines, growth factors, hormones, and invasion of bacteria (Roy et al., 2017). Although the generation of ROS and RNS is involved in crucial cell signaling functions, excessive amounts can generate malfunctions to proteins, lipids, carbohydrates, and nucleic acids, and disorders such as aging, hypertension, atherosclerosis, ischemia/reperfusion, renal diseases, diabetic neuropathies, Alzheimer’s disease and cancer (Sharifi-Rad et al., 2020; Boy et al., 2021). It is why pharmaceutical exploration aimed at modulating the oxidative response in therapies is a very active field of research (Purohit et al., 2019). Cells in tumors are particularly sensitive to oxidative stress as they commonly present higher levels of ROS due to the dysregulation of the redox balance, and excess of ROS potentially contributes to oncogenesis by oxidative DNA damage (Montero and Jassem, 2011).

The majority of ROS/RNS are hydrogen peroxide (H_2_O_2_), hydroxyl radicals (OH^•^), superoxide radicals (O_2_˙^−^), nitric oxide (NO˙), and peroxynitrite (ONOO^−^). ROS or RNS are able to activate or inactivate proteins by reacting with sulfhydryl (sulfenylation), glutathione (GSH, glutanylation), and cysteine (oxidation) groups (Jones et al., 2004; Defelipe et al., 2015). Antioxidant proteins are important tools for the control of ROS/RNS levels and conduct target-specific transduction of redox signals. The major enzymatic antioxidants include superoxide dismutase (SOD), catalase (CAT), glutathione peroxidase (GPx), glutathione *S* transferase (GST), and glutaredoxin (Grx), and operate in cooperation with thiol-redox couples to regulate ROS/RNS levels. It is worth noting that all these enzymes are ubiquitous. Six major redox couples are usually present in a cell: NADH/NAD, NADPH/NADP, cysteine (Cys)/cystine (CySS), GSH/glutathione disulfide (GSSG), peroxiredoxin (Prx)-sulfiredoxin (Srx), and thioredoxin (Trx)/thioredoxin disulfide (TrxSS). For instance, thiol systems can adjust the production of H_2_O_2_ by limiting its diffusibility and stability in each subcellular compartment, while the pKa of specific residues on proteins determines how sensitive these residues are to the available H_2_O_2_ (Thamsen and Jakob, 2011; Jacob et al., 2012). Additionally, thiol groups can in turn be modified (e.g., nitrosylation, sulfhydration, metal ion binding) allowing to act as signaling molecules to control cell function (Marino and Gladyshev, 2011).

## Metabolic abnormalities and ROS generation in cancer cells

**FIGURE 4:**
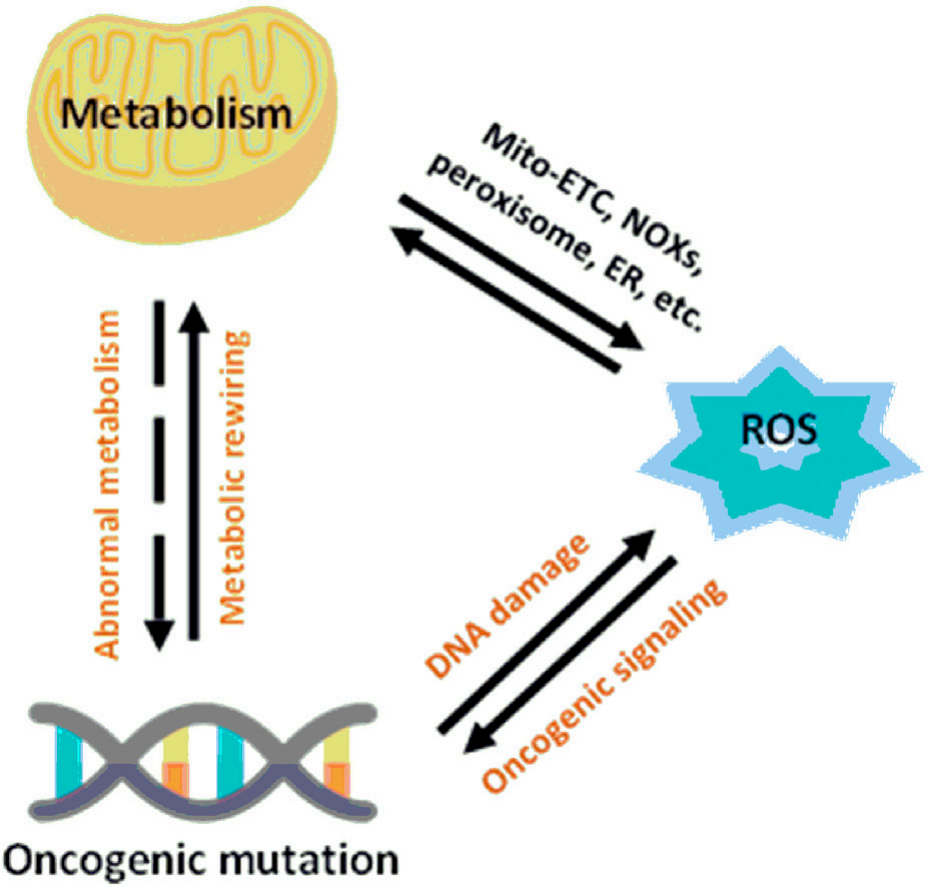
Relationship between metabolism and redox signaling in cancer cells (Wang et al., 2019).

A critical point in the metabolic–redox mechanisms in cancer is the “hypermetabolism” required for growth and proliferation of tumor cells which results in intracellular ROS production in the mitochondria, NADPH oxidases (NOXs), peroxisomes, and endoplasmic reticulum (ER) (Batinic-Haberle et al., 2011; Barrera, 2012). These mechanisms are ubiquitous, such as NOX originally described in leukocytes, but found throughout the body. NOX has seven different isoforms, NOX_1-5_, DUOX_1_ and DUOX_2_, each isoform characterized by the specific catalytic subunit, the interacting proteins and the localization in the different cells of the body (Rastogi et al., 2017). Mitochondrial ROS are byproducts of metabolic processes during which electrons escape from the mitochondrial electron transport chain (Mito-ETC) and react with molecular oxygen to generate superoxide anions (O_2_^−^) (Batinic-Haberle et al., 2012). In addition, metabolic enzymes, such as 2-oxoglutarate dehydrogenase (OGDH), pyruvate dehydrogenase (PDH), glycerol-3-phosphate dehydrogenase (GPDH), and flavoprotein-ubiquinone oxidoreductase (FQR), also contribute to O_2_^−^ production (Antunes and Cadenas, 2000; Baud and Karin, 2009; Becuwe et al., 2014). Along with Mito-ETC, oncogenic activation triggers the production of ROS through NOX-mediated NADPH oxidation (Figure 4) (Yoboue et al., 2018)

Another fundamental process for the intracellular production of ROS is the cooperation between mitochondria, the endoplasmic reticulum (ER), and peroxisomes (Wang et al., 2019).

### NADPH oxidases

NADPH oxidases (NOXs) play a fundamental role in a wide range of physiological processes, such as gene expression regulation, cell signaling and differentiation, but are also involved in many pathological processes, including cancer. Several studies have demonstrated that cancer cells often display mutations which can increase ROS generation from NOX enzymes, which in turn can lead to tumorigenesis (Jaramillo et al., 2012; Jaramillo et al., 2015). A particular type of mutation involves the GTPase KRAS, a member of the RAS oncogene family. KRAS mutations induce NOX1-mediated ROS formation and metastasis.^63-^

### Catalases

The CAT enzymes are present in most of cells exposed to oxygen and are involved in lowering high concentrations of H_2_O_2_ (Nakabeppu et al., 2006; Munro and Treberg, 2017). CAT can also react with peroxynitrite, a strong oxidizing agent produced by the reaction between nitric oxide (NO˙) and O_2_^−^, associated with pathological events (ONOO^−^/ONOOH). In cancer cells, CAT can be found in high concentrations in the plasma membrane and occasionally released in the extracellular matrix, and can act as a tumor suppressor and as a survival agent during tumor progression (Naranjo-Suarez et al., 2013; Narayanan et al., 2020). However, higher catalase levels have been associated with more aggressive cancers when compared to lower CAT concentrations (Glorieux and Calderon, 2018; Galasso et al., 2021).

### Glutathione

**FIGURE 5:**
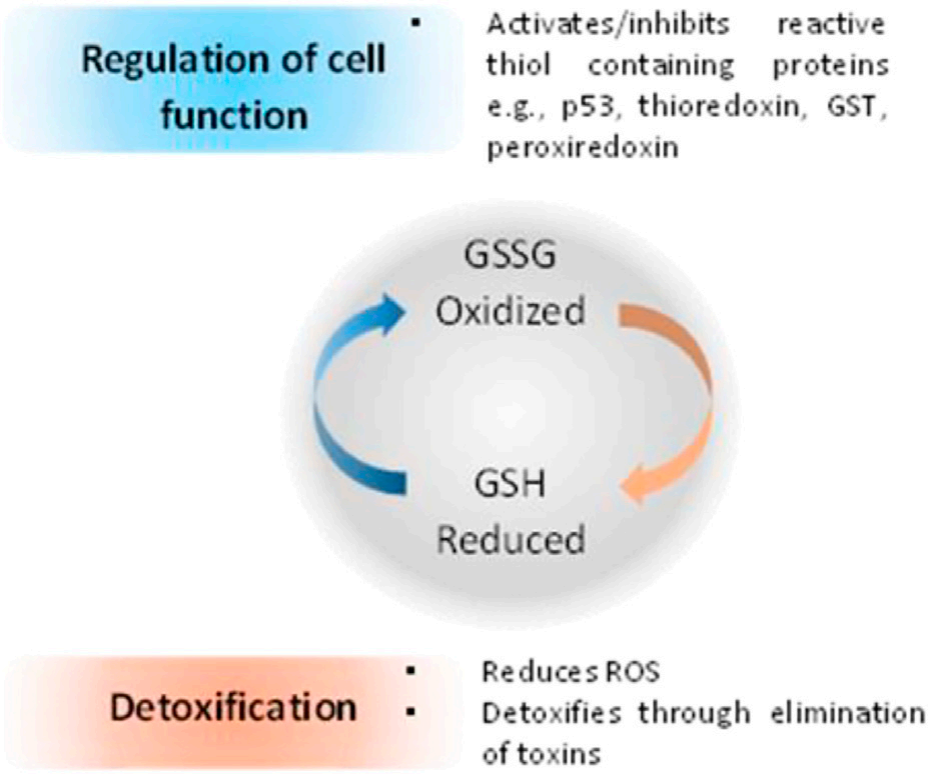
Relationship between redox state of glutathione and regulation of cell function and cell detoxification (Montero and Jassem, 2011).

When compared with normal cells, cancer cells contain higher GSH levels, as GSH metabolism appears to be involved in protecting cancer cells from apoptosis (Gamcsik et al., 2012). Furthermore, increased levels of GSH within tumor cells are associated with resistance to platinum-containing anticancer compounds, due to the formation of GSH-platinum conjugates mediated by glutathione S-transferase P1 (GSP1) (Peklak-Scott et al., 2008). The GSH related metabolism genes are regulated by Nrf2 genes, which have been used as redox state index for platinum resistant cancers (Galluzzi et al., 2012). The overall cellular redox state is regulated by three systems, two of which are glutathione-dependent: the reduced glutathione (GSH)/oxidized glutathione (GSSG) system (Figure 5), the glutaredoxin (Grx) system and the thioredoxin (Trx)/Trx reductase system (Giles, 2006; Townsend, 2007; Tew and Townsend, 2011). GSH acts directly as an electron donor, whereas Grx uses GSH or GR as an electron donor and depends on the intracellular concentration of GSH. On the other hand, Trx uses nicotinamide adenine dinucleotide phosphate (NADPH) as an electron donor, independently of GSH (Tew and Townsend, 2011).

### NADPH dehydrogenases (quinone)

Quinone reductase 1 (NQO1) can be considered the redox barrier between the organism and its environment (Oberley et al., 19801980). NQO1 detoxifies ROS-generating quinones to hydroquinones through a back and forth route, using NAD(P)H to reduce FAD and then catalyzing a two-electron reduction to generate FAD and hydroquinone (Oshikawa et al., 2010).

## Redox enzymes as a target of drugs for cancer treatment

In addition to their activity on cell division, many cytotoxic drugs are able to induce oxidative stress by modulating the concentration of ROS (Giles, 2006; Townsend, 2007). Furthermore, the susceptibility of some cancer cells towards redox enzymes has been considered as a therapeutic target for the rational design of new anticancer agents (Xiong et al., 2021). As many drugs currently applied in chemotherapies have an impact on redox pathways, probably contributing to their antitumor activity, evaluating the possibility of precisely affecting the cellular redox balance has become a leading trend in anticancer research (Table 1) (Chen and Chang, 2019)

**TABLE 1:** Redox modulation by cytotoxic anticancer drugs currently used clinically (Chen and Chang, 2019).

Target	Drug	Mechanism of redox modulation
Glutathione system	NOV-002	Induction of *S*-glutathionylation
	BSO	Glutathione depletion leading to induction of apoptosis by ROS
	TLK286	Inhibition of glutathione-*S*-transferase
	TLK199	Inhibition of glutathione-*S*-transferase
Thioredoxin system	PX-12	Inhibition of thioredoxin-1
	BNP7787	Inhibition of thioredoxin-1 and glutaredoxin
Arsenic derivatives	ZIO-101	Inhibition of catalase

Metal complexes can also affect the cellular redox chemistry, directly through metal- or ligand-based redox processes or indirectly by interacting with biomolecules implicated in cellular redox pathways (Bandeira et al., 2017; Ortega et al., 2021).

### Iron complexes

**FIGURE 6:**
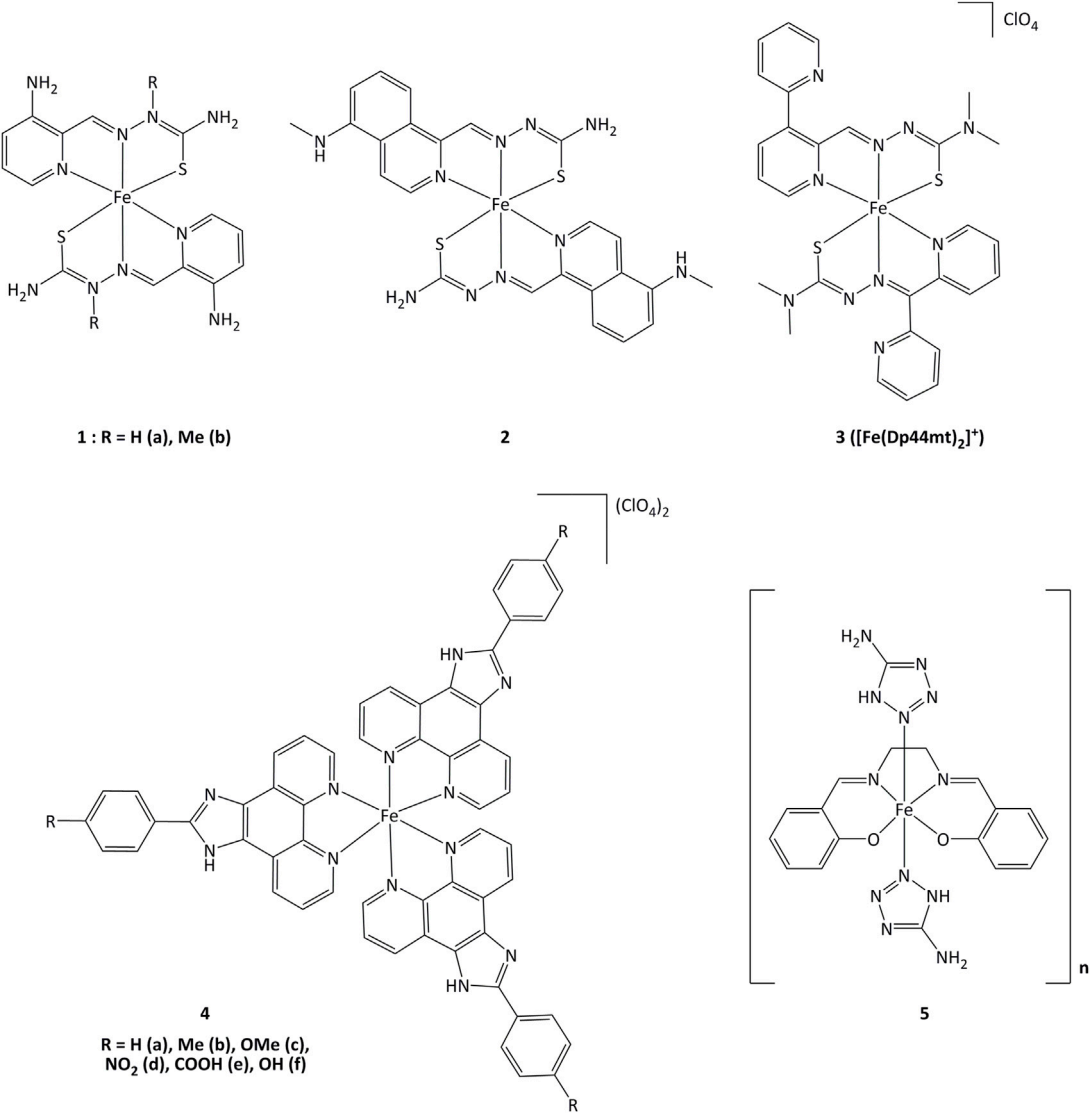
Iron complexes able to alter redox enzymatic activity.

Iron(II) complexes bearing triapine-type heterocyclic thiosemicarbazone ligands (triapine = 3-aminopyridine-2-carboxaldehyde thiosemicarbazone, a molecule studied in the treatment of cancers) have been reported to inhibit ribonucleotide reductase (RNR), an enzyme which catalyzes the reduction of ribose to deoxyribose in nucleotides for DNA synthesis (Plamthottam et al., 2019). Inhibition of RNR by triapine results in depletion of DNA precursors, selectively depriving replicating cancer cells of nucleotides for survival. The redox-active form of triapine responsible for RNR inhibition is the Fe(II) (triapine)_2_ fragment. Iron complexes with triapine analogs (**1** and **2**, Figure 6) have shown *in vitro* that redox events are crucial for RNR inhibition, and were able to inhibit cell proliferation at similar or lower concentrations (250 nM - 0.7 μM) than triapine alone (Plamthottam et al., 2019). The reductive activation of Fe(III)-triapine by thioredoxin reductase-1 (TrxR1) and glutathione reductase (GR), leading to the generation of reactive species has been demonstrated. In particular, TrxR1 displayed high activity with Fe(III)-thiosemicarbazone derivatives, and a specificity between the Fe(III) complexes and the redox centers of TrxR has been observed. (b; Myers et al., 2013; Lovejoy et al., 2011; Richardson et al., 2009). Iron(III) complex **3** (Figure 6) with thiosemicarbazone-derived ligands is reduced by ascorbate to iron(II), increasing lipid peroxidation. The formation of ascorbyl radical anion (Asc˙^−^) has been detected after adding ascorbate to the iron(III) complex, resulting in the production of ROS (Selyutina et al., 2022). The use of ascorbate to promote the redox activity of these potential anticancer agents was demonstrated *in vitro*. Complex **3** showed antiproliferative action on the human melanoma cell line SK-MEL-28 at concentrations of 3.125–25 μM, in the presence of 1,000 μM of ascorbate (Kontoghiorghes et al., 2020). Upon introduction of a methoxy group, compound **4c** displayed elevated cytotoxicity towards CaSki cancer cells. The IC_50_ values for complex **4c** were 0.75, 6.73, 7.32 and 23.71 µM for Caski, SiHa, HeLa and L02 cells, respectively. Studies on the cell death mechanisms induced by complex **4c** showed that cancer cell growth was suppressed by apoptosis, and the TrxR activity of Caski, SiHa, and HeLa cells decreased to 48.92, 84.51 and 86.01% respectively (Xie et al., 2017). Evaluation of the relationship between the inhibition of TrxR and the cytotoxic activity suggests that compound **4c** carries out its activity through TrxR inhibition, affecting cellular redox balance and leading to cell death.

Iron(III) complexes bearing salen-type ligands (salen = *N,N*′-ethylenebis(salicylaldimate) dianion) have been studied for their anticancer activity. The cell death induced by complexes like **5** was related to DNA cleavage and superoxide dismutase (SOD) mimicking activity, probably generating local imbalance in superoxide/hydrogen peroxide levels, leading to cell apoptosis. Complex **5** was highly active against K562 and MCF-7 (IC_50_ = 6.4 and 13.1 µM, respectively) with IC_50_ value of 1.89 µM for the inhibition of SOD (Herchel et al., 2009).

### Ruthenium complexes

**FIGURE 7:**
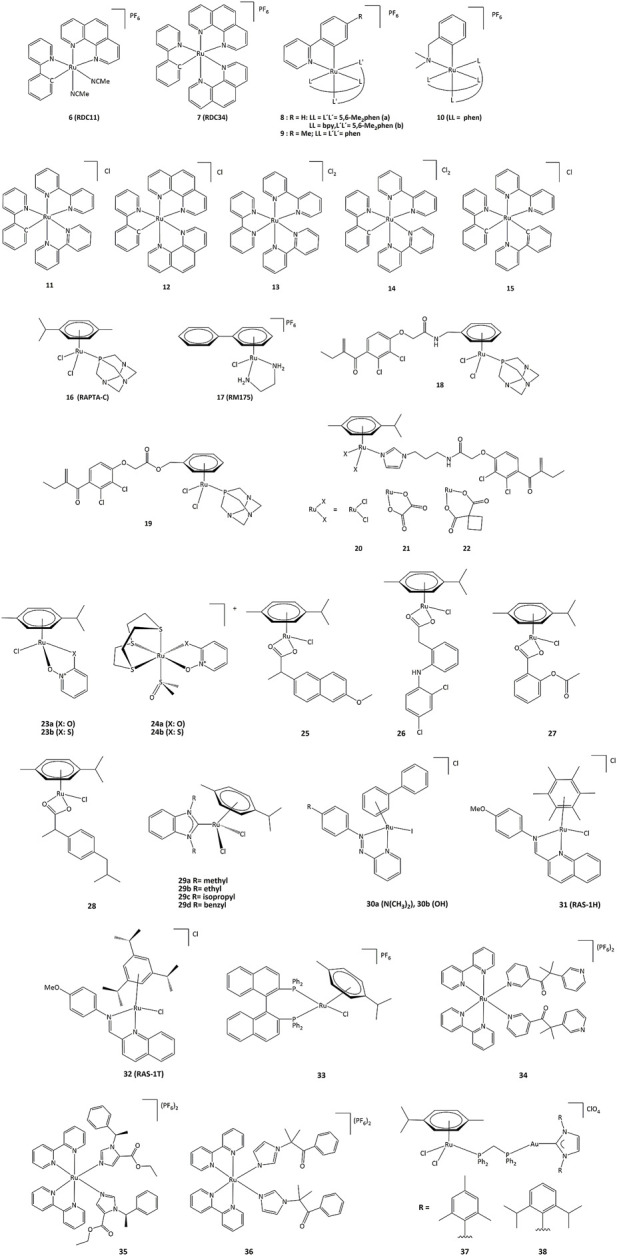
Ruthenium compounds capable to interact with redox enzymes.

Ruthenium derivatives are among the most studied and promising compounds for potential anticancer treatments. The success of ruthenium is notably due to specific redox kinetics properties and the relevant oxidation states (II) and (III). In studies aimed at the development of biosensors, our group has shown that cyclometalated ruthenium complexes can alter the activity of purified oxidoreductases, such as glucose oxidase, horseradish peroxidase, lactate dehydrogenase or PHD2 (Ryabov et al., 2001; Saavedra-Diaz et al., 2013; Bautista et al., 2016; Vidimar et al., 2019). Such compounds were used as mediators (electron shuttles) in the electron transfer to or from oxidized or reduced active sites of redox enzymes. The ruthenium complexes **7–11** shown in Figure 7 mediate the electron transfer and display high reactivity with respect to horseradish peroxidase (HRP) and glucose oxidase (GO) (Ryabova et al., 1999). Organometallic ruthenium(II) derivatives bearing cyclometalated 2-phenylpyridine (phpyH), (**11** and **12**, Figure 7), function as noncompetitive inhibitors of glucose oxidase in the oxidation of *β*-D-glucose by O_2_. The analogous coordination compound **13** behaves, in contrast, as a competitive inhibitor. Oxidation of Ru(II) to Ru(III) compounds **14** and **15** does not make the complexes competitive inhibitors (Saavedra-Diaz et al., 2013). Interestingly, if ruthenium complexes are able to inhibit redox enzyme activity, the reverse can also occur. For instance, ruthenium(III) compound **14** (oxidized form of **11)** promotes the enzymatic activity of glucose oxidase (Saavedra-Diaz et al., 2013). Bis-cyclometalated complex **15** is able to transport electrons from the reduced active site of PQQ-dependent alcohol dehydrogenase (PQQ-ADH) to an electrode with 1,2-propanediol as substrate (Le Lagadec et al., 2006). Our group also studied how modifications in the ligand structure could affect the ability of the metallacycles to interact with their direct biological targets. The activity of two purified oxidoreductases, glucose oxidase and horseradish peroxidase, was evaluated in the presence of the cyclometalated derivatives (Licona et al., 2020). The calculation of the *k*_3_ rate constant for the electron transfer between the active site of the enzyme and the complexes showed that the ability to alter the activity of both enzymes is related to their oxidoreduction potentials. The coordination of a second phenanthroline ligand in **7** (**RDC34**) lowered the redox potential by *c.a.* 100 mV and increased the lipophilicity when compared with **6** (**RDC11**). Such results showed that the modification of the spatial structure of the complexes may also be responsible for their capacity to alter the redox enzyme function (Anand et al., 2009).

To understand the role of the **RDC11** complex in cancer metabolism, studies were performed on the HIF1A (hypoxia-inducible factor) pathway (Vidimar et al., 2019). At the molecular level, **RDC11** can affect redox enzyme activities and intracellular redox state by increasing the NAD^+^/NADH ratio and ROS levels, and at the metabolic level, the HIF1A pathway is affected by inducing the activity of the iron redox enzyme PHD2, an enzyme that controls HIF1A protein levels (Vidimar et al., 2019). Notably, unlike cisplatin, the activity of **RDC11** was not affected by the presence of mutations in p53 (Gaiddon et al., 2005). As such, PHD2 could be considered a direct target of **RDC11**, which could activate the PHD2 activity through a mechanism possibly involving the redox activity of the ruthenium complex. Inhibition of HIF1A led to decreased angiogenesis in patient-derived xenografts using fragments of primary human colon tumors (Alpeeva et al., 2003).

An important feature of cancer cells is their elevated lactate production due to high glucose consumption and the switch to glycolytic metabolism. Lactate dehydrogenase (LDH), which catalyzes the production of lactate in the final step of the glycolytic pathway, is a fundamental enzyme in such process (Netanya and Robert, 2019). To get a better understanding on how cyclometalated compounds could impact on the activity of LDH *in vitro* and in cancer cells, a comparative study was performed using polypyridine ruthenium(II) complex **13** and its structurally related cyclometalated-phenylpyridine counterparts **11** and **12** (Bautista et al., 2016). The cytotoxicity in gastric and colon cancer cells induced by **11** and **12** is significantly higher when compared to 13. The inhibition mechanisms on purified LDH were evaluated and kinetic studies allowed the calculation of the corresponding inhibition constants. Though complexes 11 and 13 are structurally similar, their inhibition modes are different. Cyclometalated complex 11 behaves as a non-competitive inhibitor of LDH, suggesting no interaction with LDH in the vicinities of lactate/pyruvate or NAD^+^/NADH binding sites (Bautista et al., 2016).

Such results suggested that ruthenium complexes might affect the redox state of cancer cells by altering the activity of redox enzymes (Meng et al., 2009). This could induce the oxidation of proteins causing misfolding and activation of the unfolded protein response (UPR), also called the endoplasmic reticulum stress (ER stress) pathway (King and Wilson, 2020). The UPR pathway helps cancer cells to survive under drastic conditions and contributes to resistance in chemotherapy and radiotherapy (Limia et al., 2019). However, despite the role of UPR in promoting cancer progression and resistance to chemotherapy, artificial induction of ER stress has been suggested as a potential anticancer strategy. This approach has been successfully demonstrated with **RDC11** and **RDC34** which were able to strongly induce CHOP, a transcription factor that mediates apoptosis in response to ER stress (Meng et al., 2009). Interestingly, **RDC34** displayed a higher expression of CHOP than **RDC11**, which can be explained by a greater retention in the endoplasmic reticulum due to its higher lipophilicity (Klajner et al., 2014). Similarly, structure-activity studies of **RDC** complexes revealed that complexes with a relatively significant lipophilicity and redox potentials in a specific 0.4–0.6 V (*vs*. SCE) region were the most active. Such dependence on the redox potential probably indicates that electron transfer to/from Ru(II) should play a role in their UPR-inducing activity (Klajner et al., 2014; Gaiddon et al., 2021).

Derivatives **18** and **19** in which the arene ligand is substituted by ethacrynic acid through an amide or an ester moiety were able to inhibit GST P1-1, with IC_50_ values in the 5.9–13.7 μM range (Townsend et al., 2005; Ang and Dyson, 2006; Suss-Fink, 2010). Other ruthenium–arene complexes bearing EA-modified imidazoles (**20–22**) are also efficient inhibitors of GST P1-1 and can inhibit cells growth of cisplatin resistant human ovarian cancer cells with IC_50_ from 9 to 15 μM (Ang et al., 2007).

Complex **17** (**RM175**), which specifically binds to guanine bases of DNA, can also react with the thiol group of GSH to form [Ru(η (Nakamura et al., 1997)-biphenyl) (en) (GS]^+^ (en = ethylenediamine, GS = glutathione). Further addition of oxygen to the thiolate ligand produces the sulfenate complex. Finally, the sulfinate adduct can be generated by oxidation (Novakova et al., 2003; Wang et al., 2005). Such combination of GSH and oxidation reactions contribute to the binding to guanine in DNA. Substitution of the sulfenate ligand by guanine N7 generates a redox-mediated pathway to DNA binding (Wu et al., 2013).

The development of hormone-dependent forms of cancers of lung, larynx, and bladder cancers have been associated with isozymes from the aldo–keto reductase 1C subfamily (AKR1C) (Penning and Byrns, 2009; Lanišnik and Penning, 2014). Furthermore, AKR1C isozymes are related to the resistance to many anticancer drugs, including platinum-based (Chen et al., 2008; Chen et al., 2013). Ruthenium complexes bearing as ligands the zinc ionophores pyrithione and its oxygen-containing analog (**23–24**) have been studied against AKR1C isozymes. If compounds **23a** and **23b** were able to efficiently inhibit AKR1C1, the inhibitory activity was much lower for **24a** and **24b**. In addition, **23b** also displayed high cytotoxicity (EC_50_ = 3.8 μM) on the hormone-dependent breast cancer cell line MCF-7, when complex **24b** bearing a sulfur macrocycle was almost inactive (EC_50_ = 200 μM). On the other hand, **23a** and **24a** did not show cytotoxic effects against the same cancer cell line (Kljun et al., 2016).

Nonsteroidal anti-inflammatory drugs (NSAIDs) have been able to display chemopreventive properties in cancer cells due to their ability to block cyclooxygenase (COX-1 and COX-2) and lipoxygenase (LOX) enzymes which are often upregulated in malignant tumors (Feng et al., 2014; Banti and Hadjikakou, 2016; Boodram et al., 2016). The coordination of NSAID to a [ruthenium(arene)] moiety in complexes **25–28** allowed the inhibition of COX and LOX activity, and antiproliferative activity against series of cancer cell lines.120.

*N*-heterocyclic carbene (NHC) metal complexes have also been studied as potential metal-based drugs. For instance, ruthenium complexes **29a-29d** can react with biologically relevant thiols and selenols. TrxR enzymes activity could be inhibited by such complexes, with IC_50_ values ranging from 0.30 to 3.74 μM. The compounds are also cytotoxic against several cancer lines, with IC_50_ values of 2.06 and 51.67 μΜ for MCF-7 breast cancer cells and >100 and 2.40 μΜ for HT-29 colon cancer cells for **29c** and **29d** respectively (Oehninger et al., 2013).

The N=N azo bonds in complexes **30a** and **30b** bearing azpy-type ligands (azpy = 2-phenylazopyridine) generate redox potentials that are biologically accessible, and oxidation of GSH to GSSG is observed under physiological conditions and important levels of ROS in A549 lung cancer cells have been detected (Chow et al., 2014). Unlike RM175 which forms a key intermediate with GSH for subsequent DNA binding, ruthenium-arene complexes **30a** and **30b** can catalyze the oxidation of GSH to GSSG, with the first step being the reduction of the azo bond (-N=N-) by GSH, followed by the elimination of GSSG and the catalytic cycle is completed by the reduction of O_2_ to H_2_O_2_ and the subsequent oxidation of the ligand to regenerate the azo bond. Such ligand-based redox reactions provide new concepts for catalytic drug design (Dougan et al., 2008).

The two ruthenium Schiff base complexes, **RAS-1H** and **RAS-1T** (**31**, **32**), induced non-apoptotic programmed cell death through the ER stress mechanism (Chow et al., 2016). Interestingly, **RAS-1T** shows a ROS-mediated ER stress pathway, while **RAS-1H** is independent of ROS. However, both complexes are more active against apoptosis-resistant cell lines than clinical drugs.

Complex **33** displayed a cytotoxic activity 15 and 7.5 times higher than cisplatin against A549 and HeLa cells, respectively (Li et al., 2018). This ruthenium complex reacted with the NAD^+^/NADH couple through transfer hydrogenation reactions and also induced ROS in cells (Li et al., 2018). As overexpression of P450 enzymes in tumors is often associated with resistance to various drugs, the use of P450 inhibitors as ligands allowed the preparation of ruthenium prodrugs **34–36**, that can be triggered to controllably release the inhibitors. Activation of the compounds by light provides the free ligands that can inhibit the P450 enzymes, while the remaining ruthenium center can damage DNA (Zamora et al., 2017).

Heterobimetallic ruthenium-gold complexes **37** and **38** were highly active against series of cancer cells, displaying a better selectivity than their mononuclear counterparts. The TrxR activity of HCT116 cells was inhibited by compound **37** (IC_50_ = 5.22 µM), while cisplatin was inactive. Complex **38** presented cytotoxicity with IC_50_ values of 5.2, 73.2 and 8.1 µM towards Caki-1, HEK-293T and HTC116 cancer cells, respectively (Férnandez-Gallardo et al., 2016).

### Osmium complexes

**FIGURE 8:**
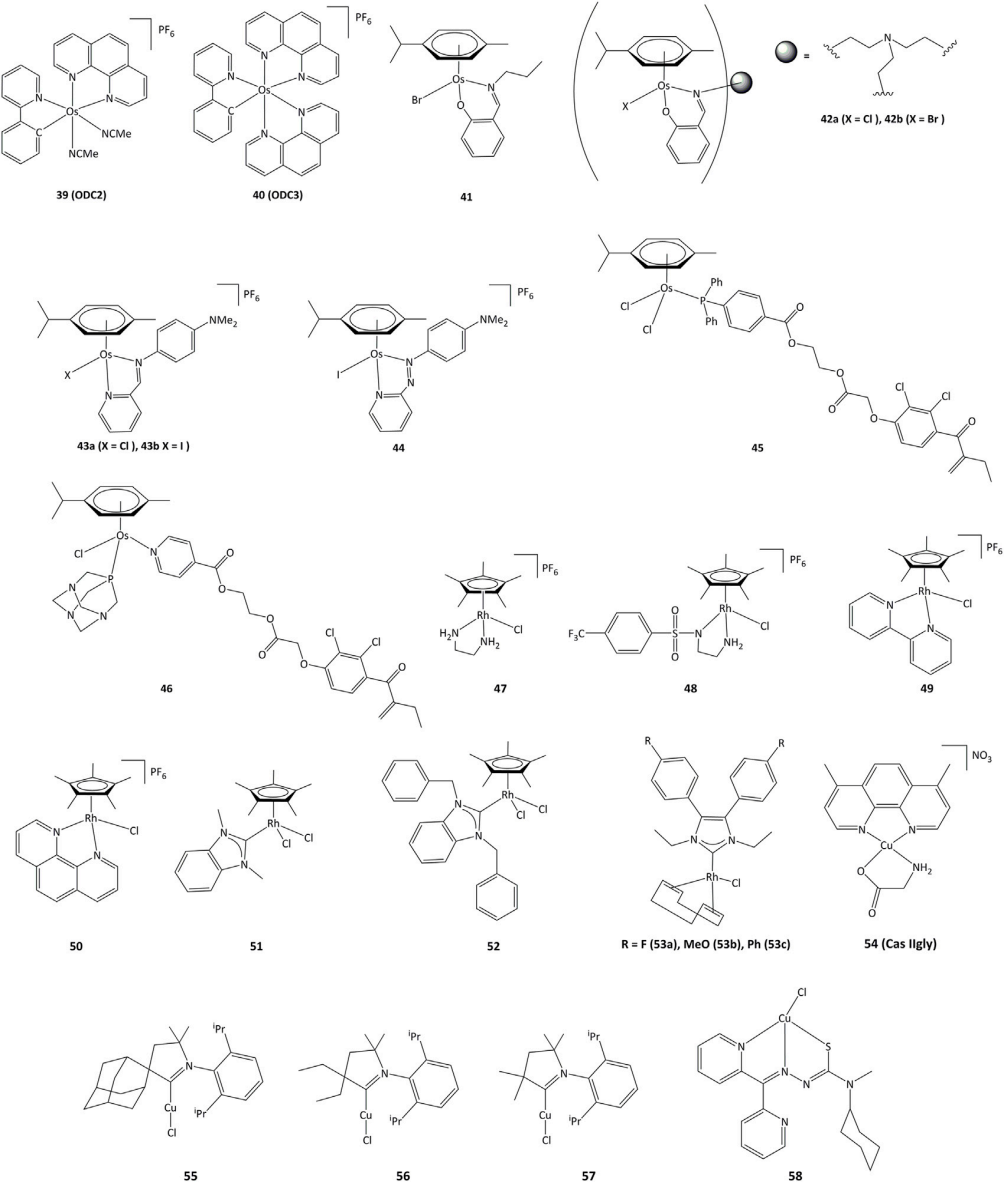
Osmium, rhodium and copper complexes studied as anticancer agents.

Cyclometalated osmium complexes synthesized by our research group have shown high cytotoxic activity, with IC_50_ below 1 μM on various series of cancer cell lines, driven by the level of lipophilicity and low reduction potential (Boff et al., 2013). For instance, the **ODC2** (**39**, Figure 8) and **ODC3** complexes (**40**) cause cell death by inducing the transcription factor CHOP and the ER stress pathway (Suntharalingam et al., 2013; Oakes, 2017; Gaiddon et al., 2021).

Mononuclear and trinuclear arene Os(II) complexes bearing pyridylimine or phenoxyimine derived ligands (**41–42**) were active against cisplatin-resistant cancer lines, and it has been shown that they were able to inhibit the topoisomerase I (Pommier, 2006; Banothile et al., 2014). The activity of related osmium complexes bearing iminopyridine ligands (**43a** and **43b**) has also been evaluated (Fu et al., 2012). Complexes **43a** and **43b** were active against ovarian and lung cancer cell lines, and their activity associated with the production of ROS and oxidation of NADH. Contrary to their ruthenium azopyridine analogues (**30a** and **30b**), the complexes cannot oxidize GSH, but can oxidize NADH to NAD^+^ through a hydride transfer to the osmium(II) center (Romero-Canelón et al., 2013). Related azo derivative **44** acts through a ROS-dependent pathway, and its cytotoxicity is inversely related to the intracellular concentration of GSH (Needham et al., 2017). Complexes **45** and **46** bearing the EA fragment were able to inhibit between 20 and 30% of GST enzyme activity, even in cisplatin-resistant cancer cell lines (Agonigi et al., 2016; Allocati et al., 2018).

### Rhodium complexes

In recent years, interest in potential rhodium(III) drugs has flourished due to their enzymes inhibition capacity (Sohrabi et al., 2021). Rhodium(III) complexes **47–50** (Figure 8) can reduce NAD^+^ to NADH using formate as the hydride source. The competition reactions between NAD^+^ and pyruvate for formate-catalyzed reduction showed a preference for NAD^+^ reduction (Soldevila-Barreda et al., 2015).

Rhodium(III) complexes bearing NHC ligands have been studied as inhibitors of TrxR. The study of IC_50_ values for various human cancer cell lines showed that the presence of a benzyl substituent on the nitrogen atoms of the NHC affected the activity, as **51** presented a lower cytotoxicity than **52** towards cancer cells. However, both complexes exhibited strong inhibition of TrxR (IC_50_ values of ∼1 μM for **51** and **52**) (Truong et al., 2020). Rhodium(I) complexes **53a-c** also showed cytotoxic activity towards MCF-7, HT-29 y HepG2 cancer cell lines, where the lowest IC_50_ values were obtained for HepG2 cells (1.33, 5.84 y 4.96 μM for **53a - 53c**, respectively). Complex **53a** was able to inhibit TrxR both *in vitro* and *in vivo* and showed an IC_50_ value of 2.5 μM for TrxR in HepG2. It is proposed that TrxR could be a possible biological target for the **53a** complex (Fan et al., 2019).

### Copper complexes

Copper plays an important role in the development of cancer, through the generation of angiogenesis and metastasis, and effective cellular uptake of copper by malignant cells has been observed (Gaur et al., 2018; Quiles et al., 2020). Copper shows high redox activity as it can easily switch between I and II oxidation states in intracellular medium, allowing potential interaction with redox enzymes. Copper(II) chelates (cassiopeins) have been evaluated as cytotoxic agents towards human lung cancer cells H157 and A549. Complex **54** (**Cas IIgly**, Figure 8) was shown to inhibit glutathione through redox cycling, generating ROS and inducing apoptosis. In both cell lines, **Cas IIgly** induced a dramatic decrease in intracellular GSH levels, most of which was oxidized to GSSG (IC_50_ values were in the 2.5–5 μM range) presumably through the reduction of Cu(II) to Cu(I) (Kachadourian et al., 2010). Cyclic(alkyl) (amino)carbene (CAAC) copper complexes **55**–**57** have been evaluated against series of cancer cells, displaying IC_50_ values around 0.14–17.4 µM on all cancer lines and TrxR inhibition of up to 52.3% at 10 μM (Bertrand et al., 2017).

Copper complexes bearing thiosemicarbazone ligands have also been studied as cytotoxic agents (Richardson et al., 2006; c; Kalinowski et al., 2009). Compound **58** allowed for a notable GSH depletion and lysosomal damage causing apoptosis, and was able to modify the GSH/GSSG ratio from 0 to 7% of the control, corroborating an important redox activity. Complex **58** showed an IC_50_ of 5 μM towards SK-N-MC cells (Lovejoy et al., 2011; Park et al., 2016).

### Platinum complexes

**FIGURE 9:**
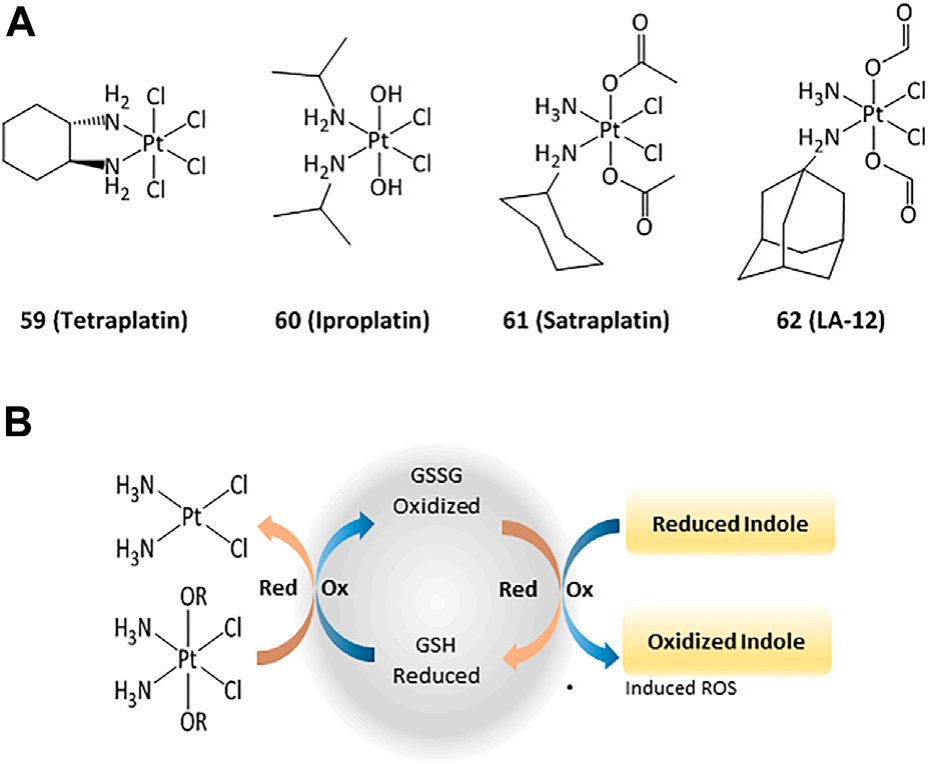
Pt(IV) anticancer drugs that have entered clinical trials **(A)** and redox interaction of Pt(IV) complexes and glutathione **(B)**.

The anticancer activity of platinum(II) complexes has generally been associated to cross-linking with the nitrogen bases of DNA, forming adducts that inhibit replication and generate strand breaks and miscoding, causing apoptosis and inhibition of RNA and protein synthesis (Ortega et al., 2021). However, DNA interactions are not the only mechanisms and targeting cytosolic proteins is also important for inducing apoptosis (Zhou et al., 2002; Ortega et al., 2021). For instance, TrxR can interact with platinum compounds and cisplatin-derivatized TrxR can provoke apoptosis in cancer cells. 4) In order to reduce the side effects and drug resistance caused by Pt(II), the use of Pt(IV) complexes has been evaluated (Hall et al., 2007). Such platinum(IV) derivatives are pro-drugs that can be reduced intracellularly to the corresponding active Pt(II) compound (Johnstone et al., 2016; Olszewski et al., 2011; e). Thus, the design of new Pt(IV) compounds displaying high cellular uptake and sensitivity to reduction by enzymes overexpressed in cancer has been highlighted (Czarnomysy et al., 2021; Zhong et al., 2020; 1; Wexelblatt and Gibson, 2012). Four octahedral Pt(IV) compounds have entered clinical trials (**tetraplatin**, **iproplatin**, **satraplatin**, and **LA-12**, Figure 9). Unfortunately, **LA-12** failed in phase I trials, while **tetraplatin** showed high neurotoxicity and was not investigated after phase I. **Iproplatin** showed limited benefits in phase II trials, and studies on the orally available **satraplatin** were dropped in phase III (Nagyal et al., 2020; Chunyan et al., 2021).

Studies on the possible routes of action of such platinum derivatives showed that the coordination of carboxylic acid ligands as redox modulators in the axial positions of the Pt(IV) center enhanced the antiproliferative effects through simultaneous DNA interactions and generation of ROS (Figure 9) (Wangpaichitr et al., 2021; Tolan et al., 2016; f)

**FIGURE 10:**
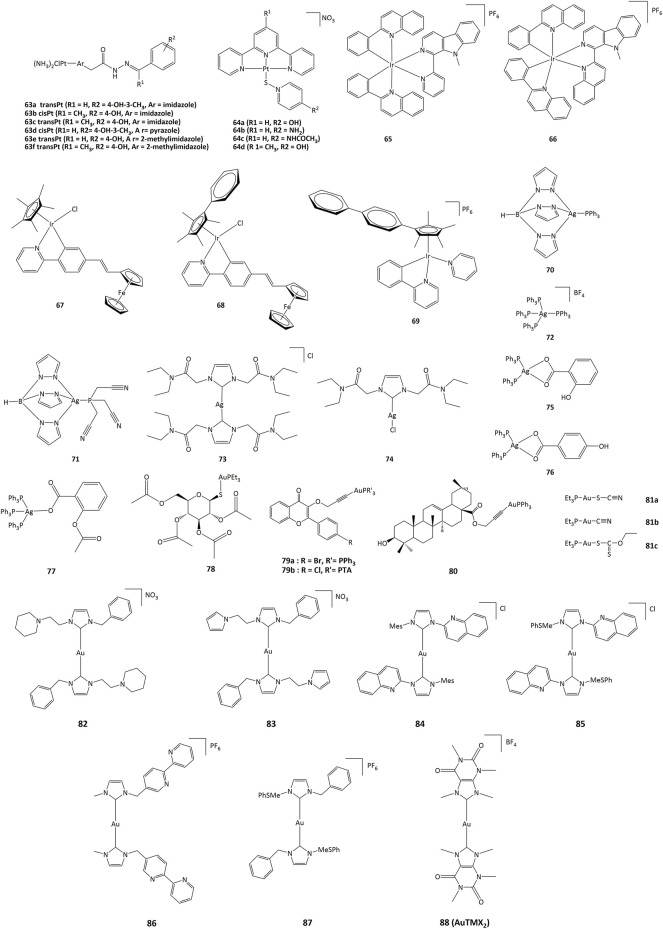
Platinum, iridium, silver and gold compounds studied as potential enzymes inhibitors.

Platinum(II) derivatives can also exhibit redox activity in biological systems. For example, cisplatin and transplatin monochlorido analogs with heterocyclic acylhydrazones (**63**, Figure 10) inhibited bovine GPx-1 and murine TrxR-1 and exhibited higher cytotoxicity than cisplatin and transplatin (Lemmerhirt et al., 2018). The IC_50_ towards various cancer cell lines were in the 0.7–22.8 μM range. Complexes **63a - 63f** exhibited higher activity than cisplatin and transplatin, with inhibition higher than 50% towards TrxR observed at 25 μM. In addition to their DNA-intercalating capacity, terpyridine-platinum(II) complexes **64** also targeted TrxR (Lo et al., 2009).

### Iridium complexes

Iridium(III) derivatives can participate in cellular redox reactions and inhibit proteins, and the use of cyclometalated ligands or the substitution of small counter-anions by larger ones allowed for the synthesis of highly cytotoxic compounds (Wang et al., 2021; g; Mou et al., 2017; Zhang et al., 2018a; Pettinari et al., 2015; Venkatesh et al., 2017) (Cao et al., 2013; Leung et al., 2013; g) (Mou et al., 2017; Prieto-Castañeda et al., 2022). The anticancer activities of such iridium complexes are generated by different mechanisms, such as catalytic interference with cellular redox balance, (Li et al., 2017), interactions with protein kinases, (Du et al., 2018), and regulation of non-apoptotic pathways (Novakova et al., 2003; Wilbuer et al., 2010). Additionally, cyclometalated iridium(III) complexes are efficient photosensitizers (PSs) capable to generate singlet oxygen (^1^O_2_), allowing for their potential application in PDT. (Lv et al., 2016; Nam et al., 2016). Other cytotoxic ROS such as superoxide anion (O_2_˙^−^) and hydroxyl radicals (˙OH) have also been produced (Novohradsky et al., 2019). The activity of cyclometalated complexes **65** and **66** (Figure 10) against lung cancer cells (A549) increased remarkably after irradiation at 425 nm, with a phototoxicity index between 93 and 120 (Qin et al., 2020). Cell-based assays showed that 66 produced a rise in intracellular ROS concentrations, reduction in ATP production, mitochondrial DNA damage, increase in lipid peroxidation, and inhibition of proteasomal activity (Qin et al., 2020).

Ferrocenyl-substituted half-sandwich iridium(III) cyclometalated-phenylpyridine complexes showed a higher cytotoxic activity than cisplatin. Notably, these bimetallic iridium–iron (**67**–**69**) derivatives were more active against A549, Hela, and HepG2 cells than their respective monometallic iridium and ferrocene compounds (Ge et al., 2019). Such activity has been explained by the easy conversion of NADH to NAD^+^ through hydride transfer by the Ir(III)Cp* group to form iridium-hydride species. The hydride can further be transferred to oxygen to form H_2_O_2_ (Lu and Holmgren, 2012; Liu et al., 2014).

### Silver complexes

The activity of silver complexes against bacteria and cancer cells can be associated to their solubility and stability in water, lipophilicity, redox properties and rate of release of silver ions (Gandin and Fernandes, 2015; Medici et al., 2016; Chenga and Qi, 2017). Homoleptic and heteroleptic phosphine silver(I) complexes **70**–**72** (Figure 10) selectively inhibit the selenoenzyme thioredoxin reductase both as an purified enzyme and in human ovarian cancer cells, with inhibition concentration values in the nanomolar range, causing disruption of cellular thiol-redox homeostasis and apoptosis (Dammak et al., 2020). Silver complexes **73** and **74** bearing NHC ligands have also been studied against series of cancer cell lines, with IC_50_ values in the range of 16–24 μM in cisplatin-resistant cells. Such silver(I) complexes also displayed TrxR inhibition with concentrations in the nanomolar range (Pellei et al., 2012).

Another series of triphenylphosphine complexes (**75**–**77**) inhibited the lipoxygenase enzyme (LOX) with IC_50_ values of 2.3, 7.6 and 7.2 µM for **75**–**77** complexes, respectively. Complex **75** presented IC_50_ values of 1.6 μM against leiomyosarcoma cells (LMS) and 2.5 μM for human breast adenocarcinoma (MCF-7). Compound **76** presented IC_50_ values of 1.6 and 2.0 μM for LMS and MCF-7, respectively, while IC_50_ for compound **77** were 1.5 and 1.6 μM for LMS and MCF-7 (Poyraz et al., 2011; Banti et al., 2012).

### Gold complexes

Among gold complexes, auranofin (**78**, Figure 10), is of special importance. Auranofin was approved by the FDA for the treatment of rheumatoid arthritis in 1985 and is currently evaluated for applications in neurodegenerative diseases, acquired immunodeficiency syndrome, parasitic and bacterial infection, as well as anticancer agent. The routes of action of gold compounds often involve enzyme inhibition, and the anticancer activity of auronafin has mainly been attributed to the inhibition of TrxR enzyme (IC_50_ = 82.6 nM) (Marzano et al., 2007).

Gold(I) complexes **79** bearing flavone-derived ligands displayed anticancer activity towards undifferentiated Caco-2 and MCF-7 cells with IC_50_ values lower than cisplatin and similar to auranofin. The IC_50_ values for compounds **78** and **79** towards undifferentiated Caco-2 cancer cell line were 1.52 and 2.33 μM, respectively. The cytotoxicity of complexes **79** can be associated with the inhibition of cyclooxygenase 1/2 enzyme and alteration of the activity of thioredoxin reductase and glutathione reductase (Mármol et al., 2019). Gold(I) complex **80** with oleanolic acid-derived ligand can provoke apoptosis in ovarian cancer A2780 cells through different mechanisms, such as induction of ER stress and inhibition of TrxR. Gold(I) compounds bearing pentacyclic triterpene ligand are able to inhibit the TrxR enzyme with an IC_50_ value of 2.61 μM, while free pentacyclic triterpene showed an IC_50_ > 50 μM. Complex **80** was active against A2780 cells, with IC_50_ of 10.24 μM (Mianli et al., 2020).

Linear gold(I) complexes bearing triethylphosphine and cyanate (**81a**), thiocyanate (**81b**) or ethylxanthate (**81c**) ligands were able to inhibit TrxR1 and TrxR2. The IC_50_ values towards TrxR1 were 1.1, 1.8 and 0.7 nM, and 7.8, 5.0 and 3.6 nM for TrxR2, for **81a**—**81c**, respectively. Complexes **81a**—**81c** presented IC_50_ values of approximately 80 and 2-fold lower than those of cisplatin and auranofin, respectively, towards different cancer cell lines such as HCT-15 (IC_50_ = 0.32, 0.08 and 0.61 μM for **81a-c**) and HeLa (IC_50_ = 0.18, 0.09 and 0.13 μM) (Gandin et al., 2010). On the other hand, gold(I) NHC complexes **82**–**87** were active against A2780cis, A2780, HepG2, HepAD38 and MDCK cancer cell lines with IC_50_ values in the 0.11–5 μM range (Schuh et al., 2012; Hickey et al., 2008; Karaca et al., 2017; h). The cytotoxic activity of these compounds has been associated to the ability to generate cell cycle arrest through different pathways, particularly via the inhibition of TrxR. The cellular activity of TrxR was reduced by about 55–60% in A549 lung cancer cells upon treatment with 2.5 μM of the gold derivatives (**82**–**87**) (Zhang et al., 2018b).

Recently, Gerner *et al.* showed that the cationic bis-NHC gold(I) complex [Au(9-methylcaffeine-8-ylidene)_2_]^+^ **88**) can display multimodal activity in ovarian cancer cells. It was demonstrated that **88** affects nuclear and telomeric proteins. It also affects actin, leading to the induction of Nrf2 genes, in parallel with the production of GSH. Treatment of cancer cells with **88** also led to a 2-fold reduction in the ratio of reduced to oxidized glutathione (Meier-Menches et al., 2020).

## Conclusions

In this review, we highlighted recent developments on the use of transition metal complexes as anticancer agents acting through changes in the intracellular redox balance and interaction with redox enzymes. The tuning of the redox properties of the complexes through the rational design of the ligands and judicious choice of metal and oxidation state is crucial for their ability to interact with redox active enzymes, resulting in increased biological and anticancer activities. Although the exact mechanisms of action for the cytotoxicity exerted by such metal derivatives are not always unquestionably determined, evaluating the roles played by redox interactions provides essential information that would allow to prepare more effective and selective antineoplastic drugs. To reach this goal, an extensive effort has to be taken using unbiased approaches (proteomic, transcriptomic, metabolomic) to compare the activity of a wide range of metal complexes and identify the direct interactants, regulated pathways and metabolites that are impacted by those compounds. Such methodology will allow to decipher without bias the physico-chemical determinants that drive the cytotoxicity and redox impact of metal complexes on cells. In parallel, biologists and oncologists need to further elucidate how cancer cells adapt to the metabolic challenges raised by tumor growth, aiming at identifying novel druggable targets for metal-based molecules.
